# Universal health coverage, economic slowdown and system resilience: Africa’s policy dilemma

**DOI:** 10.1136/bmjgh-2017-000400

**Published:** 2017-08-30

**Authors:** Giuliano Russo, Gerald Bloom, David McCoy

**Affiliations:** 1 Centre for Primary Care and Public Health, Queen Mary University of London, London, The United Kingdom; 2 Institute of Development Studies, The University of Sussex, Brighton, The United Kingdom

**Keywords:** Universal Health Coverage, health financing, Africa’s economy, health system’s resilience, health systems in LMICs

**Summary box**

- Achieving universal health coverage (UHC) has become a dominant global health policy preoccupation during the last decade, advocating ambitious healthcare coverage goals, increases in health funding and financial pooling mechanisms for social protection;
- As many commodity-dependent African economies are presently experiencing a marked slowdown and international assistance is becoming more volatile, there seems to be a growing divide between UHC principles and policy-makers’ everyday concerns in the field;
- In order to keep inspiring health development in Africa, UHC thinking and international health support need to take into account the continent’s non-linear growth pattern and the need to ensure that its health systems are resilient to external shocks;
- Drawing from past mistakes and from the continent’s reaction to past crises, a number of macro, meso and micro policies can be identified to strengthen the UHC concept, and reconcile its aspirations with Africa’s current economic outlook.

## Introduction

Achieving universal health coverage (UHC) has become a dominant policy preoccupation within the global health community. For Africa, progress towards UHC involves ambitious goals for expanding access to a range of effective health services, a substantial increase in health expenditure, and establishing a greater reliance on prepayment and pooling mechanisms to finance healthcare.^1^ According to one set of calculations, achieving UHC requires countries to spend at least $86 per capita in 2012 dollars on healthcare, and a minimum of 5% of Gross Domestic Product (GDP).^2^ Clearly, expanding the ‘fiscal space for health’^3^ will be key to the success of UHC.^i^

The global UHC movement is welcome and has helped to galvanise political will to tackle the problem of growing health inequities and the impoverishing effect of out-of-pocket health expenditures.^4^ It also helped refocus attention on the fragmented and inefficient architecture of domestic and international health financing,^5^ the unpredictability of foreign aid^6^ and the lack of regulation over the private health sector in low- and middle-income countries (LMICs).^7^ The concept of health insurance has become central to the promotion of UHC, in the belief that financial and risk pooling offers the best guarantee for cost-effective expenditure and protecting the most vulnerable from financial hardship.^8^ Public financing will need to play a critical role,^9 10^ and it has been argued that domestic taxation should be designed to both expand the fiscal space for health and pursue social justice objectives.^11^

However, the promotion of UHC in Africa has been largely built on the optimism associated with the continent’s commodity boom and improved economic performance over the past decade. As the economic outlook for the continent deteriorates, optimism about the viability of ambitious UHC policies and targets is beginning to recede. This paper argues that if the continent is to remain committed to the concept of UHC, the associated policies and financial tenets need to incorporate the nature of Africa’s non-linear, fragile growth.^12^

## Africa's economy and financing in times of slowdown

For the last decade, Africa’s development has been propelled by high prices for export commodities, the discovery and exploitation of natural resources, new-found stability and governance improvements in key countries and the ripening demographic dividends.^13^ However, following the collapse of world prices for oil and other minerals, growth in sub-Saharan Africa decelerated to 1.5% in 2016, the lowest level since the 1980s, with the region’s real GDP per capita contracting by 1.1%; South Africa and oil-exporting countries accounted for most of the region’s slowdown.^14^ A recent International Monetary Fund report predicts a bleak outlook due to a dramatic drop in the price and volume of exports and falls in employment for 28 sub-Saharan African countries.^15^ Natural disasters, localised armed conflicts and the Ebola epidemic in west Africa have only compounded an already difficult situation. Although African economies are diverse and include 24 lower and upper middle-income countries according to the World Bank,^16^ the impact of the economic slowdown will be felt in many parts of a continent that is still heavily dependent on export commodities and vulnerable to political instability.

This is not the first time that Africa has had to cope with serious financial constraints. In the late 1970s and the 1980s, many sub-Saharan Africa’s countries were hit hard by the combination of high oil prices (which was then imported) and low prices for key export commodities, compounded by recurrent natural disasters and political instability.^17^ This led to economic recession, unsustainable debt and the imposition of austerity programmes that devastated public services. That negative economic cycle was overcome, also on the account of foreign direct investment in the continent and remittances from its diaspora.^18^ However, back then, mistakes were made; structural adjustment programmes and health sector reform policies were designed and implemented under the guidance of development partners. The general consensus is that these programmes and policies were largely ill-conceived and impacted negatively on the development of many of Africa’s fragile economies and nascent public services.^19^ In the health sector, these prolonged crises were all associated with falls in the value of public salaries and health workers numbers,^20^ frequent drug shortages and neglect of infrastructure and equipment maintenance. The introduction of user fees and community-based financing schemes did not alleviate the impact of insufficient public funds,^21^ with long-lasting regressive effects on population health.^22^

Past experiences also illustrate the different coping strategies that institutions and individuals adopted to weather the economic storm and bounce back. In Mozambique, prioritising essential primary care services and protecting personnel and generic drug expenditures helped protect the country’s health system during its civil war years.^23^ A focus on infectious diseases, essential drugs and context-specific primary health care (PHC) policies were the steps South Africa took to protect the health system from apartheid era distortions.^24 25^ Across the continent in the 1980s and 1990s, health professionals started selling public drugs to compensate for reductions and delays in their salary payments.^26^ In Malawi, international partners stepped in to support the top-up and payment of health worker salaries in the public sector.^27^ And many patients were forced to rely on their personal savings, remittances and borrowing to guarantee continued access to services.^28^

As well as facing the challenges of economic contraction, declining public budgets and political instability, many of the poorer countries on the continent presently have to contend with the changing priorities of donors and aid programmes (box 1).

Box 1 **Shifting environments and disrupted health financing strategies in three low-income African countries**

In postconflict Sierra Leone in 2001, the national government and international donors embarked on an ambitious Free Health Care Initiative (FHCI) to remove user fees from the provision of healthcare to pregnant/lactating women and under-5s. The Ebola epidemic in 2014 and the global economic slowdown have come to jeopardise the initiative. A recent review^27^ concluded that there are currently not enough domestic resources to pay for the requirements of the FHCI, or universal health coverage (UHC), and that increased donor support will be needed for the next decade.

In Mozambique, the dip in commodity prices, a resumption of armed confrontation, a severe drought in parts of the country and the discovery of hidden government debts have all recently dented confidence in the country economy. While the government and development partners had been content to develop a sustainable health financing strategy up until 2015, inspired by the aim of UHC, the discovery of the hidden debts has resulted in international donors suspending aid and loans to the country^28^. Although the aid-dependent health sector has been granted an additional 10% in public funds to compensate for the withdrawal of foreign assistance, the plans to strengthen the country’s health financing system have been all but put on hold.

In Guinea-Bissau, most donors suspended direct contributions to the State budget in 2014 following the latest coup d’état, leaving the chronically underfunded health sector susceptible to the problems of illegal charges and health workers routinely misappropriating revenues from the sale of medicines.^29^ Similar to what happened before in other countries,^25^ the World Bank temporarily agreed to support public health salaries, with the objective of avoiding public servants’ strikes, supporting modernisation of the public administration and stabilising a volatile situation. With the end of this support and collaboration with the national government still suspended, international health partners are negotiating health financing arrangements directly with non-governmental organisations to allow the provision of basic health services in the country.

## The tensions between universal health coverage (UHC) and Africa's non-linear economic growth

With the prospect of economic contraction and shrinking budgets, many African countries will face great challenges in reaching the ambitious goals of UHC. How should they respond? Of course, achieving UHC has always implied the need for a medium-term to long-term plan to strengthen health systems and ensure sustainable, equitable and effective health financing policy.^29^ But there is a need to consider how the health system can withstand and be resilient in the face of an economic slowdown or contraction while still striving to expand access and services.

By resilient, we mean health actors, institutions and populations being able to maintain core functions and maintain good health when a crisis hits, and draw from the lessons learnt during the crisis to reorganise.^30^ Resilience also implies an ability to draw on personal resources to face adverse circumstances, ensure effective prioritisation and protect core functions that will allow to bounce back when the crisis is over.^31^ To build up resilience, measures are needed to reduce population’s exposure to infections, preventing common infectious diseases and ensuring effective surveillance mechanisms. Action is also needed to protect the population against dangerous practices and counterfeit drugs, and to provide them with reliable, trustworthy information and advice. These are some of the core public health functions that should define any health system, but that resources-poor governments often neglect, particularly during an economic slowdown.^32^ Furthermore, in some settings, informal providers and a variety of private practitioners happen to deliver a large proportion of healthcare, particularly to the poor^33 34^; in a resilient health system, these coexist with formal health services in a regulated way that provides training, monitoring, supervision and technical support, and enables informal providers to earn a living. Policies are needed to bring the different ways health services are provided in resource-scarce settings under the broad vision of UHC.

## Squaring the circle of Africa's health policy dilemma

The goal of UHC should still apply in times of economic slowdown. If anything, there is even more of a need to ensure universal access to essential healthcare in times of economic crisis. But policies must incorporate the realities of non-linear economic growth and potential economic contraction. While solutions to Africa’s political and macroeconomic instability are important, they lie beyond the scope of this commentary. And while we recognise the fact that the impact of the regional economic crisis will be uneven across the continent, we argue that past experiences point to the general need to consider a certain set of health sector-specific policies.

At a macro level, efforts must be made to keep expanding the fiscal space for health in both low-income and middle-income African countries. Crises also often present unexpected windows of opportunity to access extra resources for health, reform health systems, adopt unusually bold actions and take on ingrained special interests for the greater good.^35^ Because of the increased leverage of international funds during an economic crisis, donors could be more effective in negotiating earmarked windfalls from natural resources for social sectors, increased budget allocations for the health sector, reforms to make them more progressive introducing health-related levies and mobilising extra international assistance. At the regional governance level, the establishment of Africa’s Centres for Disease Control and Prevention is another example of how to improve the continent’s capacity to identify its own epidemiological issues and solutions to strengthen its health systems.^36^

At the meso (sector-wide) level, learning from the deleterious consequences of the Structural Adjustment Programmes in the 1980s,^37^ countercyclical measures should be brought in to mitigate the effects of the crisis on population health and health systems, and provide social protection for low-income and vulnerable groups. This is likely to be more feasible for those middle-income countries with wider fiscal space; as shown in Cuba,^38^ investments made at decentralised and district level of health administration and local health communities have the potential to boost system resilience.^39^ The introduction of National Health Insurance schemes should be piloted in phases to ensure the programmes are resilient to economic downturn, only to be scaled up in the following expansionary phases. During economic contractions, recurrent expenditures (paying for salaries, drugs and basic maintenance) often take precedence over capital ones.^35^ However, African governments could still find ways to maximise resources and reduce costs by moving away from wasteful input-based to performance-based financing,^40^ reducing out-of-pocket financing for vulnerable populations by eliminating user fees, introducing solidarity funds and contracting out services. Private providers and private resources should be brought into the UHC equation to be regulated and harnessed to improve their quality and avoid dangerous distortions,^41^ but also as a recognition of their importance as essential coping mechanisms that individuals and health systems fall back to when everything else fails.

At the micro (health programme implementation) level, priority should be given to preventive primary services as well as to the procurement and distribution of basic drugs, and to retain key personnel; public health programmes and surveillance mechanisms should be strengthened against communicable diseases to avoid possible epidemics.^32^ Ensuring funding for salaries and basic drugs will have to take precedence over setting up complex pooling arrangements, as well as conducting minimum infrastructure and equipment maintenance interventions to avoid irreversible deterioration. The spread of recent technological advances such as mobile telephones and finance may create opportunities for the introduction of more cost-effective interventions such as telemedicine, thus strengthening the existing systems.^42^

UHC principles have helped move forward Africa’s health systems for the past decade. Learning from past mistakes and from recent advances in health financing theory, national governments and international health partners can now adapt such principles to the continent’s current slowdown, identify policies to build resilient health systems and protect core functions and services for its population. The issues relating to health system resilience are to be given important considerations while developing policies for achieving UHC irrespective of the context, since different countries are at different stages in their progress towards achieving UHC.

## Footnotes

i ‘Fiscal space’ refers to the ability of national governments to raise revenues for their health objectives within the existing macroeconomic constraints, taking into account their long-term sustainability.^3^
